# Active Recovery between Interval Bouts Reduces Blood Lactate While Improving Subsequent Exercise Performance in Trained Men

**DOI:** 10.3390/sports5020040

**Published:** 2017-06-12

**Authors:** Harutiun M. Nalbandian, Zsolt Radak, Masaki Takeda

**Affiliations:** 1 Graduate School of Medicine, Kyoto University, Kyoto 660-8501, Japan; 2 Department of Physiology, University of Physical Education, Budapest 1123, Hungary; radak@tf.hu; 3 Faculty of Sports and Health Science, Doshisha University, Kyotanabe 610-0394, Japan; mtakeda@mail.doshisha.ac.jp

**Keywords:** acidosis, high-intensity interval training, recovery, Wingate test

## Abstract

This study aimed to examine the blood lactate and blood pH kinetics during high-intensity interval training. Seventeen well-trained athletes exercised on two different occasions. Exercises consisted of three 30 s bouts at a constant intensity (90% of peak power) with 4 min recovery between bouts followed by a Wingate test (WT). The recoveries were either active recovery (at 60% of the lactate threshold intensity) or passive recovery (resting at sitting position). During the exercise, blood samples were taken to determine blood gasses, blood lactate, and blood pH, and peak and average power were calculated for the WT. When performing the active recovery trials, blood pH was significantly higher (*p* < 0.01) and blood lactate was significantly lower (*p* < 0.01) compared with the passive recovery trials. WT performance was significantly higher in the active recovery trials: peak power was 671 ± 88 and 715 ± 108 watts, and average power was 510 ± 70 and 548 ± 73 watts (passive and active respectively; *p* < 0.01). However, no statistically significant correlations were found between the increased pH and the increased performance in the active recovery trials. These results suggest that active recovery performed during high-intensity interval exercise favors the performance in a following WT. Moreover, the blood pH variations associated with active recovery did not explain the enhanced performance.

## 1. Introduction

The effects of active recovery (i.e., low intensity exercise) between high-intensity interval training (HIIT) have been the focus of much research. While some studies have reported that active recovery improves performance in subsequent exercise [1,2,3,4], others have shown that passive recovery is the best option [5,6,7]. The majority of the studies that have reported positive results for active recovery have had longer recoveries periods in comparison with their counterparts. A faster phosphocreatine resynthesis [2,5] and a faster pH recovery have been proposed as the main causes of enhanced performance when active recovery is complete [8]. However, most of the studies in which the effects of active recovery are evaluated are based on “all out” exercise (e.g., Wingate test (WT)), so active recovery may be affecting the exercise protocol itself. One of the main causes for fatigue has been argued to be muscular acidosis (i.e., decrease in pH). Some studies still associate the decline in power production during high-intensity exercise with a decrease in intracellular pH [9,10,11]. During high-intensity exercise, large amounts of lactate and H^+^ (hydrogen proton) are produced inside the muscle cells. Whether pH plays a role in exercise fatigue or not, it is highly regulated during exercise, and the lactate production, transport, and metabolism work as a buffer system reducing the concentration of H^+^ in cells [12]. Lactate is produced mainly in glycolytic fibers and co-transported with an H^+^ through monocarboxilate transporter 4 (MCT4). During exercise, most of the blood lactate is co-transported with one H^+^ to the inside of the oxidative fibers through the monocarboxilate transporters 1 (MCT1) and serves as fuel for the oxidative phosphorylation [13,14,15].

Lactate and H^+^ are two molecules that are highly transported into the blood stream, particularly during exercise. The aim of this study was to observe if active recovery between high-intensity bouts affects performance, and to study the blood pH, blood bicarbonate, and blood lactate kinetics.

## 2. Materials and Methods

### 2.1. Participants

A total of 17 healthy, well-trained, active young men (mean ± standard deviation, age 20.6 ± 1.9 years, weight 69.3 ± 6.7 kg, height 174.3 ± 5.7 cm, body fat 13.4 ± 1.6%, and VO_2_max 59.5 ± 5.8 mL·min^−1^·Kg^−1^ of free fat mass) agreed to participate in this study. Participants were members of different university sport clubs (rugby and baseball), with at least 4 years of training experience and were regularly training as minimum 4 sessions per week. Importantly, when classified according to the practiced discipline, the subjects did not show significant differences in terms of VO_2_max. All subjects were previously informed of the experiments and associated risks, and signed consensus documents were obtained. All experiments designed in this study, as well as the informed consent documents, were approved by the Local Research Ethics committee and are in strict accordance with the standards set by the Declaration of Helsinki (Doshisha University ethical committee, approval number: 15033).

### 2.2. Protocol

All exercises were performed in a leg cycle ergometer (MONARK 874 E, Stockholm, Sweden). During the tests, the respiratory gas exchange variables were continuously measured (breath by breath) with a Jaeger Oxycon Pro Jaeger (Wuerzburg, Germany), and the power was obtained continuously from the pedal frequency and saved in a computer for posterior analysis. The room temperature was set at 22 °C.

### 2.3. Preliminary Tests

Before the main test, the subjects completed two weeks of familiarization exercises, which consisted of a two weekly one-hour session of high-interval exercise in a cycle ergometer. After the familiarization period, the VO_2_max and the lactate threshold (inflection point in the lactate accumulation curve) from the subjects were determined using a continuous incremental test, which consisted of cycling at >60 rpm with an initial workload of 60 W and an incremental of 30 W every two minutes until volitional exhaustion was reached. Respiratory gas exchange variables were measured breath by breath, and a 30 s average was used for the determination of VO_2_. Blood lactate concentration was obtained every 2 min from capillary blood to determine the lactate threshold (lactate pro two; ARKRAY Co., Kyoto, Japan).

During a separate day, subjects performed a WT (Wingate test) that consisted of cycling for 30 s at the maximal voluntary frequency with a workload of 0.75% body weight [16].

### 2.4. Main Tests

**Figure 1:**
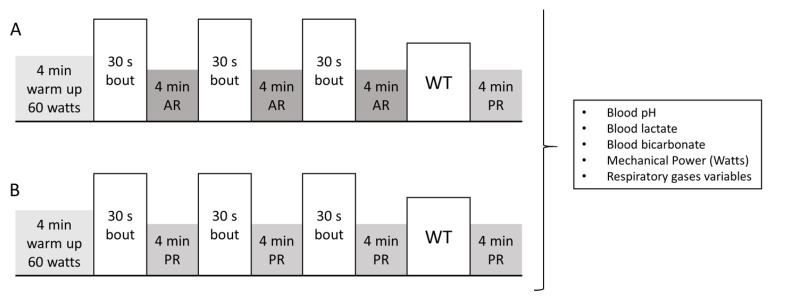
Main test exercise protocol. 30 s: pedaling at 90% of peak power. Active recovery (AR): 4 min of active recovery pedaling at 60% of lactate threshold (**A**). Passive recovery (PR): resting at sitting position. WT: Wingate test (**B**).

For the two main tests, each subject reported to the laboratory at the same time of day in order to prevent diurnal variations in performance. The two visits were separated by one week and the testing order was random. Subjects were instructed to repeat the same meals the days before the tests, and to fast for two hours before the stipulated start time. Before the tests, a standardized warm up was carried out on the cycle ergometer, and after the warm up and in a lapse of 5 min, a catheter was injected in an ante-cubital vein. The main test consisted of three 30 s bouts at constant intensity with 4 min of recovery between each bout (either active recovery; AR, or passive recovery; PR each occasion), followed by WT and a 4 min PR (Figure 1). The intensity of the 30 s bouts was set at 90% of peak power (measured in a previously done WT) at a pedal frequency of 90 rpm, and the intensity of the AR was set at 60% of the lactate threshold at 60 rpm (most efficient intensity for blood lactate clearance [17,18]). Five seconds before the 30 s bouts, subjects were asked to increase pedal frequency to the 30 s bout frequency, but without workload. Power output was recorded from every 30 s bout and shown in real time in order to determine if the intensity was falling down. In case the pedal frequency falls lower than 85 rpm for 5 s, the test would be annulated. AR and 30 s bout pedal frequency were indicated by the sound of a metronome. Fifteen seconds before the WT subjects stopped pedaling and set the pedals in horizontal position and get ready to start. Power output was recorded during WT, and average- and peak-power were calculated for each subject. Additionally, oxygen consumption (VO_2_), carbon dioxide production (VCO_2_), and respiratory exchange ratio (RER) were measured breath-by-breath during the test. For the analysis of these variables, the exercise protocol was divided into 4 phases (each phase represents the average of one 30 s bout and the following 4 min recovery, the last phase includes the WT and the last 4 min of PR).

During the tests, blood samples were taken from ante-cubital vein via catheter, and blood lactate from capillary blood using a lactate pro two (ARKRAY Co., Kyoto, Japan): before and immediately after every 30 s bout and WT, as well as 4 min after the WT. Blood samples were immediately refrigerated at 4 °C and within 40 min analyzed in a blood gas analyzer (SYSMEX, Kobe, Japan, model: OPTI CCA TS) to determinate blood pH, and blood bicarbonate and respiratory gas exchange variables (VO_2_, VCO_2_, and RER) were measured breath by breath and averaged every 30 s for posterior analysis.

### 2.5. Statistical Analysis

Statistical analyses were realized using IBM SPSS Statistic version 22.0 for Windows (IBM SPSS Co. Chicago, IL, USA). Before any statistical analysis was carried out, assumptions of normality were verified with the Kolmogorov–Smirnov test. To evaluate the effects of the two different trials (active and passive recovery) on blood gas variables, blood lactate, and respiratory gas exchange variables, a two-factor repeated measures ANOVA with Bonferroni post-hoc test was used. To evaluate the performance in the two different instances, a two-tailed paired Student’s *t*-test was used. To evaluate possible correlations between performance and measured variables Pearson’s correlation coefficient was used. Data are expressed as mean value ± standard deviation, and the *p*-values were accepted as statistically significant at *p* < 0.01.

## 3. Results

Statistical differences were found between trials when comparing performances in the WT (average and peak power both with *p* < 0.01), and these results are shown in Table 1 and expressed in mean ± standard deviation. Fifteen out of 17 subjects had a higher peak power with the AR, and 16 had a higher average power with the AR. With AR, performance increased by a 6.6 ± 1.4% and 5.3 ± 0.9% (peak power and average power, respectively). Additionally, power averages for the first 10 s and last 20 s were calculated. AR performance was 7.8% and 4% higher in the first 10 s and last 20 s, respectively (these differences were non-statistical significant).

**Figure 2:**
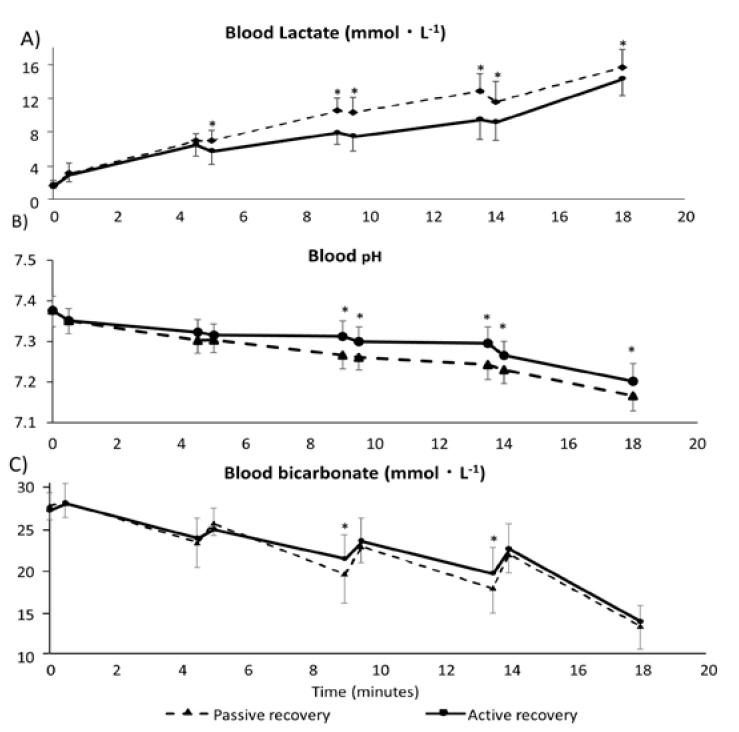
Blood lactate, blood pH, and blood bicarbonate during the main tests. (**A**) Blood lactate (mmol/L), it was significantly higher during the PR trials, and in both conditions significantly decreased during the last two 30 s bouts and the WT. (**B**) Blood pH, it was gradually decreased during the whole exercise, and statistical differences were found between conditions for measurements after the first 4 min recovery. (**C**) Blood bicarbonate (mmol/L), it was significantly different between the two trials: before the third 30 s bout and before the WT. Black bars indicate standard deviation. * *p*-values lower than 0.01.

For blood lactate (Figure 2A), statistically significant differences were found between the two trials. Bonferroni post-hoc analysis revealed that the lactate was significantly lower with the AR trial (*p* < 0.01 for values after the second 30 s bout). In both conditions, blood lactate significantly decreased during the last 30 s bouts and the WT. The lactate accumulation during the WT was of −1.3 ± 2 mmol/L and −0.3 ± 1.4 mmol/L (AR and PR, respectively), while during the 4 min after the WT was of 5.1 ± 3.1 mmol/L and 4.1 ± 3 mmol/L (AR and PR, respectively).

Blood pH decreased during the exercise, and statistical differences were found between conditions (Figure 2B). Bonferroni post-hoc analysis revealed statistical differences (*p* < 0.01) for measurements after the second 4 min recovery. Blood bicarbonate also presented significant differences between the two trials; Bonferroni post-hoc analysis showed statistical differences: before the third 30 s bout and the WT (*p* < 0.01). Blood bicarbonate decreased during and increased after the 30 s bouts and the WT (Figure 2C).

**Figure 3:**
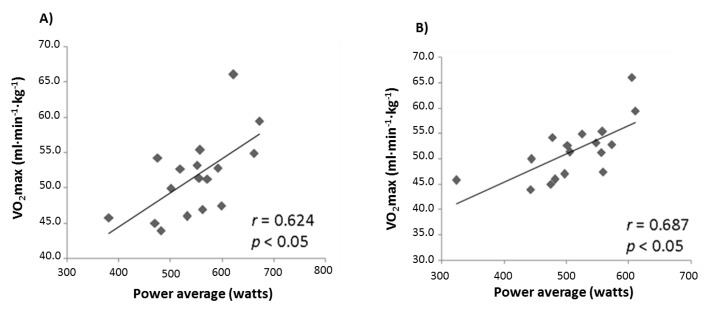
Correlations between average power in the WT for the active recovery trial (**A**) and the passive recovery trial (**B**).

Correlation analyses were performed for the differences in performance between conditions and the differences in blood pH at the moments before the WT. Furthermore, VO_2_max measured in the incremental test was correlated with performance in the WTs (Figure 3). No statistically significant correlations were found between AR and PR blood pH difference values at the moment before the WTs, or between AR and PR WT performance scores. VO_2_max was significantly correlated with the WT performance (peak and average power) for both trials.

Respiratory gas exchange variables data are shown in Table 2. VO_2_ and VCO_2_ were significantly higher in the four phases when AR was performed. RER was significantly lower in Phases 1–3, but significantly higher in Phase 4 when AR was performed. Additionally, the VO_2_ average during the test significantly increased in a time-dependent manner when comparing phases (*p* < 0.01). Overall means for the gas exchange variables were, for VO_2_ (mL·min^−1^), 1782 ± 316 and 1113 ± 102; for VCO_2_ (mL·min^−1^), 2173 ± 154 and 1539 ± 83; and for RER, 1.23 ± 0.17 and 1.36 ± 0.09 in the active and passive trials, respectively.

## 4. Discussion

The objectives of this study were to observe if the enhanced performance due to active recovery was related with the blood lactate and blood pH. Our major findings were that lactate concentration significantly decreased during the high-intensity bouts, and that changes in blood pH were not correlated with increases in performance when AR is performed.

Though blood pH was significantly higher when AR was performed, the increase in blood pH at the moment before the WT was not correlated with differences in performance (neither peak power nor power average). The decrease in pH during exercise has long been argued to be one of the main causes of muscular fatigue during high-intensity exercise [19,20,21]. The present findings indicate that the differences in blood pH do not reflect improvements in the performance when AR is performed during HIIT. As blood pH is related to muscle pH, this result suggests that pH variations are not responsible for enhanced performance. Furthermore, it can be argued that there are other factors that influence performance, such as an increased blood flow due to previously performed exercise, which may facilitate oxygen supply to the muscle for a faster phosphocreatine resynthesis [2,5,22]. This may explain why the main differences in performance were observed in the first 10 s of the WT (7.8% increased performance with AR), a period in which phosphocreatine plays an important role in energy production.

During HIIT, the lactate production rate is substantially increased. In the present study, it was observed that blood lactate significantly decreased during the WT (1.3 ± 2 mmol/L and 0.3 ± 1.4 mmol/L in AR and PR, respectively), which suggests the following hypothesis: blood lactate decreases, hence the lactate muscle uptake rate is higher than the release rate during HIIT. This phenomenon was also reported in thoroughbred horses during 2 min high-interval training [23], where blood lactate decreased during 2 min intervals, but the same did not occur when the intervals were of 5 and 15 min. Additionally, it has been reported that, when the WT is performed without fatigue, 20% of the consumed energy comes from the aerobic metabolism [24], and when a second WT is repeated after 4 min, the aerobic metabolism contribution becomes 49% [25]. Supporting this, we showed a significant correlation between the VO_2_max of the subject and their performance in the WT (Figure 3), which suggests that aerobic capacity is a determinant of repeated high-intensity exercise modalities. Taking into account the contribution of aerobic metabolism during high-intensity exercise and our data, we can hypothesize that lactate is an important energy source during repeated high-intensity exercise. In the future, further research must be done to clarify whether or not this energy source may have a determinant role in high-intensity exercise performance.

Another result of this study was that significant differences were found between AR and PR for respiratory gas exchange variables (VO_2_, VCO_2_, and RER). A predominating AR aerobic metabolism is therefore expected to have higher levels of VO_2_ and VCO_2_, and lower RER. After the WT (in both conditions), the recovery was passive, but the VO_2_ and CO_2_ were higher and the RER was lower after the PR trial, which may be explained by an increased rate in the metabolism of lactate caused by the accumulation of blood lactate. Additionally, VO_2_ average during phases increased in a time-dependent manner, which is coherent with the hypothesis that an increase in aerobic metabolism compensates the reduction in energy supply from anaerobic pathways during repeated high-intensity exercise.

These results suggest that AR during HIIT has beneficial effects in consecutive high-intensity exercise performance and requires a higher overall oxygen consumption. Moreover, slight variations in blood pH do not correlate with increased performance when AR is performed during HIIT. It might be, therefore, that active recovery should be considered for inclusion in training programs. According to our data, performance and energy expenditure would increase.

## 5. Study Limitations

This study had two main limitations. First of all, we did not label the lactate, so we did not prove that lactate is highly metabolized during high-intensity exercise. Secondly, we could not measure muscle pH and nor muscle lactate during the experiments. It is true that blood lactate and blood pH reflect their muscle contra-parts, but further research is needed to confirm if these phenomena are also taking place in the muscles.

## Figures and Tables

**Table 1:** WT performance in both trials. Values are expressed in mean ± standard deviation.

Trial	Peak Power (Watts)	Power Average (Watts)
Active	715 ± 108 *	545 ± 73 *
Passive	671 ± 88	517 ± 70

* significant differences from the Passive trial (*p* < 0.01).

**Table 2:** Respiratory gas exchange variables for each trial. Values are expressed in mean ± SD.

Variable	Trial	Phase 1	Phase 2	Phase 3	Phase 4
VO_2_ mL·min^−1^	Act.	1805 *	1954 *	2039 *	1332 *
	Pass.	1080	1025	1170	1257
VCO_2_ mL·min^−1^	Act.	2130 *	2228 *	2305 *	1985 *
	Pass.	1589	1436	1510	1622
RER No unit	Act.	1.18 *	1.14 *	1.13 *	1.49 *
	Pass.	1.47	1.40	1.29	1.29

* significant differences from PR (*p* < 0.01).
